# Categorizing Extrachromosomal Circular DNA as Biomarkers in Serum of Cancer

**DOI:** 10.3390/biom14040488

**Published:** 2024-04-17

**Authors:** Enze Deng, Xiaoying Fan

**Affiliations:** 1 Guangzhou National Laboratory, No. 9 XingDaoHuanBei Road, Guangzhou International Bio Island, Guangzhou 510005, China; 2 GMU-GIBH Joint School of Life Sciences, The Fifth Affiliated Hospital of Guangzhou Medical University, Guangzhou Medical University, Guangzhou 510005, China

**Keywords:** extrachromosomal circular DNA, biomarker, serum, cancer

## Abstract

Extrachromosomal circular DNA (eccDNA), a double-stranded circular DNA molecule found in multiple organisms, has garnered an increasing amount of attention in recent years due to its close association with the initiation, malignant progression, and heterogeneous evolution of cancer. The presence of eccDNA in serum assists in non-invasive tumor diagnosis as a biomarker that can be assessed via liquid biopsies. Furthermore, the specific expression patterns of eccDNA provide new insights into personalized cancer therapy. EccDNA plays a pivotal role in tumorigenesis, development, diagnosis, and treatment. In this review, we comprehensively outline the research trajectory of eccDNA, discuss its role as a diagnostic and prognostic biomarker, and elucidate its regulatory mechanisms in cancer. In particular, we emphasize the potential application value of eccDNA in cancer diagnosis and treatment and anticipate the development of novel tumor diagnosis strategies based on serum eccDNA in the future.

## 1. Introduction

EccDNA stands as a pivotal manifestation of circular DNA elements, representing a dynamic landscape in genomic architecture [1]. Within the intricate tapestry of genetic materials, eccDNA emerges as a notable entity, demonstrating its significance as a mechanism of somatic genome copy number amplification [2]. The taxonomy of eccDNA delineates three distinct classes, comprising intricate, small extrachromosomal circular DNA (which is known as circular microDNA, derived from a unique nonrepetitive sequence, and enriched in the 5′-untranslated regions of genes, exons, and CpG islands [3]); expansive, large, copy-number-amplified extrachromosomal circular DNA (which ranges from an arbitrary lower limit of 1 kb up to several Mb, representing thousands of genes, leading to oncogene amplification and serving as a powerful driver of intratumoral heterogeneity [4,5,6]); and enigmatic rings and/or neochromosomes (which are amplified, rearranged, and corroded through hundreds of cycles, leading to the overexpression of oncogenes [7]). Each of these contributes to the rich mosaic of genomic diversity. An unprecedented surge of scholarly attention has been directed towards unraveling the intricacies of eccDNA, particularly in the field of oncology [8]. The burgeoning body of literature dedicated to eccDNA illuminates its multifaceted roles in gene regulation [9], genome stability [10], and cancer evolution [11], underscoring its paramount importance in shaping the molecular landscape of cellular function and behavior.

In the realm of biomedical research, biomarkers play a crucial role as indicators of biological processes, disease progression, and treatment efficacy by providing insights into the underlying molecular mechanisms of cancer development and progression, as well as other disorders [12,13,14]. Therefore, the identification and validation of reliable biomarkers hold significant promise for improving cancer diagnoses, prognoses, and therapeutic outcomes. Biomarkers encompass a diverse array of molecules, genes, proteins, or other measurable substances that can be detected and quantified in biological samples [15]. In recent years, circulating cell-free DNA (cfDNA) has garnered a significant amount of attention as a biomarker for various cancers. Numerous studies have highlighted elevated serum cfDNA levels among cancer patients, with correlations established between these levels and prognosis as well as disease activity [16]. These studies indicate that in certain scenarios, alterations in cfDNA are detectable prior to cancer diagnosis, suggesting their potential as biomarkers to monitor the occurrence of cancer [17]. Circulating cfDNA has emerged as a rapid and noninvasive biomarker, providing crucial complementary information for the diagnosis, prognosis, and treatment stratification of cancer patients [18].

In recent years, mounting evidence suggests that eccDNA plays a crucial role as a cancer biomarker [19]. Consequently, it has become imperative to systematically categorize and elucidate the molecular mechanisms of various eccDNA species within the realm of cancer biomarkers. This review seeks to provide a comprehensive synthesis of the diverse roles played by eccDNA in cancer, delving into its potential significance in the diagnosis and treatment of cancer. By elucidating the complex interplay of eccDNA in the context of cancer, we aim to attain valuable insights for the development of more effective diagnostic and therapeutic strategies, thereby advancing our understanding of the molecular intricacies inherent to cancer biology.

## 2. Research Progress of eccDNA in Cancer

**Figure 1:**
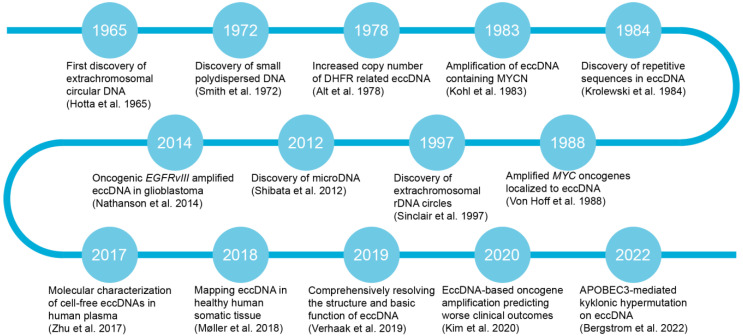
Timeline of key historical milestones in the discovery of eccDNA [3,20,21,22,23,24,25,27,28,29,31,32,33,35].

Since the initial discovery of eccDNA in neuroblastoma cell lines in 1965 [20], subsequent studies have revealed the presence of small polydisperse circular DNA [21] and identified repetitive sequences within eccDNA [22]. In 1978, Alt et al. made a pivotal discovery by identifying the presence of eccDNA in mouse cells and elucidating the mechanism behind *Dhfr* gene amplification, which conferred methotrexate resistance to the mouse cells [23,24]. A reevaluation of large-scale whole-genome sequencing (WGS) datasets has revealed the presence of eccDNA in a subset of tumors across various cancer types [25]. Moreover, oncogenes such as *MYCN*, *MYC*, and *EGFR* have been observed to undergo amplification, a phenomenon that is localized to eccDNA structures [26,27,28,29]. After these discoveries, researchers uncovered extrachromosomal rDNA circles and microDNA, expanding our understanding of the diverse landscape of eccDNA structures [3,30,31]. Zhu et al. conducted a study on the molecular characterization of cell-free eccDNA in human plasma, aiming to enhance our understanding of their molecular mechanisms and potential clinical applications [32]. In normal human muscle and blood cells, tens of thousands of eccDNAs have been observed, with many carrying intact genes or fragments [33]. Bergstrom et al. identified a novel clustered somatic mutation phenomenon termed kyklonic hypermutation, which is frequently detected on eccDNA, enhancing our understanding of the complex dynamics underlying tumor progression and heterogeneity [34,35]. Some eccDNA entities exclusively harbor enhancers without associated genes, suggesting that tumor cells containing eccDNA can exploit the enhancer function of eccDNA to drive oncogene expression [36]. Overall, Figure 1 provides a timeline of the key milestone discoveries in eccDNA research, reflecting the advancements and discoveries in this field.

## 3. Formation of eccDNA

**Figure 2:**
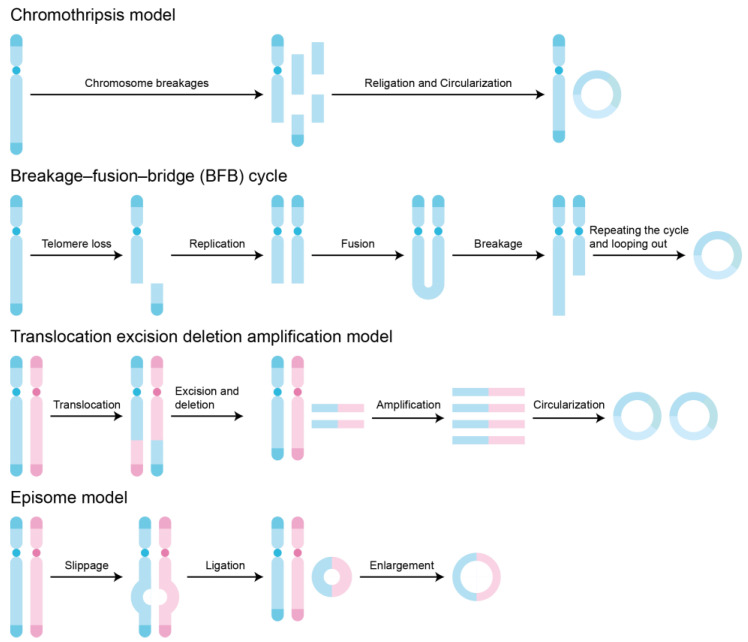
The diverse mechanisms of eccDNA formation.

The formation of eccDNA is a complex and diverse process involving various mechanisms and pathways. Although the exact mechanisms underlying its generation remain incompletely elucidated, researchers have proposed several different formation models to explain its pathways (Figure 2). The chromothripsis model describes a drastic chromosomal shattering event in which multiple regions of a chromosome undergo simultaneous breakage and rearrangement [37]. This fragmentation may lead to the circularization of chromosome fragments, ultimately forming eccDNA. This model explains why, in some cases, eccDNA carries genetic information from multiple different chromosomal regions. The breakage–fusion–bridge (BFB) cycle model involves chromosome breakage, fusion, and bridging processes [38]. In this model, the broken ends of chromosomes may fuse, forming a circular structure. Subsequently, this circular structure may be stretched into a bridge during cell division, further breaking and rearranging to generate eccDNA. The BFB cycle is an iterative process that may result in the gradual accumulation of eccDNA. In the translocation–deletion–amplification mechanism process, certain regions of a chromosome may undergo translocation, fusing with other chromosomal regions [2]. These fused regions may then undergo deletion and amplification, eventually forming eccDNA. This model explains why eccDNA may carry genetic information that is different from that of the original chromosome. In the episome model, eccDNA is viewed as autonomously replicating extrachromosomal genetic elements [39]. They may originate from certain regions of chromosomes, detach from the chromosomes via unclear mechanisms, and independently replicate in the cytoplasm. These autonomously replicating eccDNAs may be distributed to daughter cells during cell division, thereby maintaining their stability within the cell population. These models provide a foundation for understanding the importance of eccDNA in the fields of biology and medicine. It is important to note that these models are not mutually exclusive; in fact, multiple mechanisms may concurrently exist during the formation of eccDNA. Future investigations should aim to uncover the interactions and regulatory networks among these mechanisms.

## 4. Detection and Quantification of eccDNA

Microscopic imaging techniques have played a pivotal role in the history of eccDNA research. Initially, scientists identified circular DNA structures in mammalian cells through observations made with electron microscopy, providing the first visual evidence of eccDNA [20]. Subsequently, researchers discovered high-molecular-weight eccDNA when examining the karyotypes of surgically excised tumor tissues. Under optical microscopy, these eccDNAs appeared as small double-stained bodies in metaphase cells stained with DNA dyes [40]. With the continuous advancement of microscopic imaging techniques, we are now able to depict the structure of eccDNA with a high resolution using techniques such as structured illumination microscopy and atomic force microscopy [41]. These high-resolution images provide detailed information about the morphology, size, and distribution of eccDNA, aiding in a deeper understanding of its function and behavior within cells [42]. Moreover, Yi et al. utilized a CRISPR dCas9-based DNA labeling system to successfully achieve a dynamic visualization of eccDNA in live cells [43]. By designing single guide RNAs targeting specific breakpoints of eccDNA, they could introduce fluorescent tags into eccDNA and track their uneven segregation during mitosis. This technology not only provides a new means to observe dynamic changes in eccDNA within cells but also helps elucidate the mechanisms underlying eccDNA’s roles in cellular proliferation and genetic information transmission.

Through an analysis of WGS data, it is possible to reconstruct eccDNA. In the genomic landscape of tumors, regions with extreme amplifications and rearrangements exhibit inconsistent paired-end and split-read counts, indicative of circular DNA. To infer and resolve the structures of these circular DNA, bioinformatics analysis tools such as AmpliconReconstructor have been developed [44]. CReSIL is a robust computational tool designed to detect and map both simple and chimeric eccDNA from long-read sequencing datasets [45]. In addition, eccDNA can also be identified through the reanalysis of ATAC-seq data using tools such as Circle_finder [46]. Other high-throughput techniques, especially epigenetic technologies such as ChIP-seq, PLAC-seq, ATAC-seq, MNase-seq, 4C-seq, and Hi-C, are also valuable for exploring the topological structure and function of eccDNA [47]. These techniques have been used to reveal the chromatin accessibility and nucleosome compression status of eccDNA, enabling the distinction between transcripts originating from genes on eccDNA and those from linear chromosomes [30]. However, these bioinformatic algorithms often overlook a significant proportion of low-frequency eccDNA. To address this limitation, the Circle-seq technique has emerged. Circle-seq is a purification and detection method specifically designed to screen for new or low-abundance eccDNA within the genome [48]. CIDER-Seq is another novel method for detecting circular DNA, based on the random primer amplification of circular DNA followed by long-read single-molecule sequencing [49]. Long-read sequencing technology can overcome the limitations of short-read sequencing methods in accurately resolving the complex structures of eccDNA. Fan et al. introduced SMOOTH-seq, adopting long-read sequencing to resolve eccDNA at the single-cell level [50]. In addition, Chang et al. devised scGTP-seq to simultaneously detect genomic and transcriptomic alterations [51], and Chamorro et al. described scEC&T-seq, which enables the simultaneous sequencing of eccDNA and mRNAs from single cells [52]. These multiomics sequencing methods shed light on intercellular variations in eccDNA content while exploring their structural diversity and transcriptional consequences.

With the rapid development of high-throughput sequencing and bioinformatics analysis techniques, we have been able to confirm the widespread presence of eccDNA in human diseases. This discovery underscores the importance of establishing databases related to eccDNA to systematically annotate and functionally analyze these DNA elements. In response to this need, Zhao et al. constructed a public database called CircleBase [53]. CircleBase is specifically designed for the annotation and functional analysis of eccDNA in various human cells, featuring highly interactive visualization capabilities. Meanwhile, Peng et al. also constructed a human cancer eccDNA spectrum database named eccDNAdb [54]. This database not only offers basic information and annotations regarding eccDNA, but also provides data on the prognostic value of eccDNA genes. Guo et al. developed TeCD, a comprehensive platform designed for users to search and access eccDNA data while analyzing potential functions associated with eccDNA [55]. The eccDNA Atlas, a user-friendly database of eccDNA developed by Zhong et al., offers a high-quality and integrated resource for browsing, searching, and analyzing eccDNA across multiple species [56]. The establishment of these databases related to eccDNA provides researchers with powerful tools to systematically investigate the role and mechanisms of eccDNA in human diseases.

## 5. EccDNA-Related Alterations in Cancer

**Figure 3:**
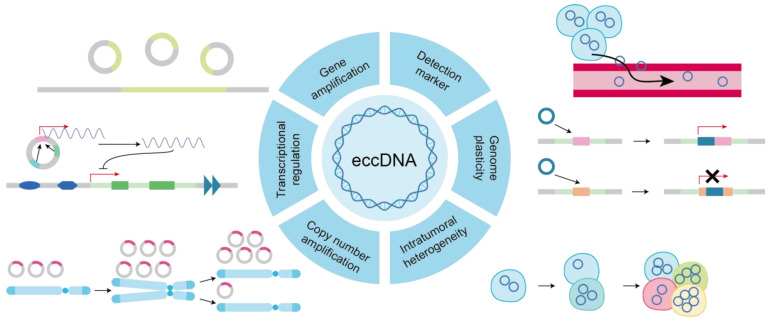
The multifaceted functions of eccDNA in oncogenesis. The heterogeneous distribution and replication kinetics of eccDNA throughout the cell cycle contribute to its uneven segregation and eccDNA-specific replication mechanisms, promoting intra-tumoral heterogeneity and elucidating its regulatory function in maintaining genomic stability and modulating cancer cell behavior.

The quantity of eccDNA molecules varies between cells, implying an uneven segregation of eccDNA during mitotic phases [23]. Notably, eccDNA lacks centromeres and is prevented from being properly distributed even during mitosis by spindle apparatus forces during the middle phase of the cell cycle. Upon the initiation of DNA replication, eccDNA molecules relocate from the nuclear periphery to the center, suggesting the existence of eccDNA-specific replication mechanisms [57]. During mitosis and segregation, eccDNA appears to hitchhike by preferentially binding to the telomere regions of linear chromosomes. Sister eccDNA molecules migrate to the same daughter cell during mitosis, which may indicate the presence of post-replicative mechanisms, such as eccDNA tethering, facilitating their physical separation [58]. Moreover, liberated from chromosomal position constraints and the lack of centromeres, eccDNA unevenly segregates into daughter cells, facilitating rapid increases in copy number and driving intra-tumoral heterogeneity [43]. These dynamics underscore the intricate regulation of eccDNA distribution and replication throughout the cell cycle, revealing potential insights into the mechanisms governing genomic stability and heterogeneity in cancer cells. Additionally, the random segregation of eccDNA during cell division results in the heterogeneity of cancer cells [59] (Figure 3).

The abundancy of eccDNA molecules in cancer cells can rapidly fluctuate in response to the ever-changing environment [60]. The combination of uneven segregation and the competitive advantage provided by the overexpression of oncogenes can lead to an accelerated amplification of clones containing eccDNA, which are sometimes observed with hundreds of eccDNA copies within one single nucleus [61]. Adaptive responses have been observed in patient tumors, where subclones containing eccDNA rapidly contract under targeted therapy but reappear upon the removal of the treatment pressure [62]. The dynamic ability to decrease and increase eccDNA levels under stress conditions may be particularly effective, potentially including factors encountered in the tumor microenvironment, such as hypoxia and high acidity levels [63]. The epigenetic state is linked to stress responses and can facilitate transient, site-specific copy number increases at loci like the *EGFR* gene, particularly in the context of extrachromosomal DNA [64]. The amplification of oncogenes in eccDNA affords selective growth advantages to cancer cells [65,66], and eccDNA also alters the layout of regulatory elements to guide the transcription of oncogenes [67,68]. Relative to chromosomal regions, eccDNA is more prone to acquiring activating mutations, further contributing to positive selection [4,69,70]. Such adaptability not only reflects the dynamic nature of eccDNA but also accentuates its singular contribution to molding the genetic terrain of tumors.

Recent evidence underscores the distinctive chromatin architecture of eccDNA compared to linear DNA within chromosomes [71]. EccDNA manifests as an increased chromatin accessibility and establishes long-range chromatin contacts, setting it apart from its chromosomal counterparts [36]. This heightened chromatin accessibility within eccDNA amplicons is further corroborated by elevated signal densities observed in circularized and amplified gene loci [72]. Furthermore, frequent amplifications of genes pertinent to tumorigenesis are recurrently observed in eccDNA [73]. These genetic features endow eccDNA with the potential to instigate malignant transformations, thereby driving tumor evolution and warranting its consideration as a prospective biomarker for tumor diagnosis and prognosis [74,75].

## 6. Relationship between EccDNA and Tumor Progression

During tumor progression, the association between eccDNA and cancer offers an alternative avenue for clinical diagnosis and treatment. Primarily, in the realm of early tumor diagnosis and detection, eccDNA emerges as a reliable biological feature and effective biomarker [76]. Secondly, in the clinical study of tumors, therapeutic strategies targeting eccDNA-driven tumor resistance hold promise for enhancing treatment efficacy [25]. Thirdly, in the prognostic phase of cancer, eccDNA serves as a potent tool for predicting patient outcomes [77]. Alterations in the eccDNA landscape can reflect the advanced metastatic stages of cancer. For instance, the lower expression of DNMT1^circle10302690–10302961^ in both primary and metastatic tumors has been associated with a poorer prognosis in cases of high-grade serous ovarian cancer [8]. Thus, the investigation of eccDNA holds significant potential in the various stages of cancer diagnosis, treatment, and prognosis assessment.

The accumulation of eccDNA within cells exerts a significant impact on the malignant phenotype of tumors. It is noteworthy that patients with a higher abundance of eccDNA containing oncogenes exhibit significantly lower 5-year survival outcomes, indicating a correlation between the abundance of oncogene-containing eccDNA and the aggressiveness of tumors [71]. Computational models further reveal that tumors harboring circular amplicons display higher cellular proliferation scores and lower immune infiltration scores [78]. Computational analyses of WGS data conducted by Kim et al. revealed that circular amplicons of eccDNA are more prevalent in aggressive cancers such as glioblastoma multiforme [25]. These findings underscore the potential of eccDNA as a key contributor to the aggressive behavior of tumors and highlight its relevance in shaping the malignant phenotype.

EccDNA emerges as a significant contributor to various cancer types, playing multifaceted roles in cancer pathogenesis and progression. Serving as a potential adjunct diagnostic and prognostic biomarker, eccDNA harbors oncogenes that undergo amplification, further fueling tumorigenesis [79]. For instance, in colorectal carcinoma, the eccDNA-mediated amplification of *DHFR* contributes to the development of drug resistance mechanisms [80]. Similarly, in ovarian cancer, eccDNA-associated factors such as *MYCN* and *EIF5A2* modulate the expression of eccDNA via MARS elements, highlighting the intricate regulatory mechanisms orchestrated by eccDNA in disease pathogenesis [81]. Additionally, the eccDNA amplification of *HER2* in breast cancer contributes to tumor resistance to therapeutic interventions [82]. Collectively, these findings underscore the diverse and pivotal roles of eccDNA in driving oncogenesis and tumor progression in various malignancies.

In addition, several studies have highlighted the efficacy of eliminating eccDNA in tumors to mitigate oncogene amplification, thereby ameliorating the malignant phenotype of tumors. Ambros et al. discovered that the extrachromosomal amplification of *MYCN* copies could be eliminated in neuroblastoma, despite their initial presence within the nuclei of flat cells [83]. As the amplification sequences within flat cells diminished, their proliferative activity correspondingly decreased, while the expression levels of major histocompatibility complex class I (MHC I) increased. A previous study revealed the mechanism of leukemia by examining the gradual loss of unstably amplified *DHFR* genes in eccDNA when HL-60 cells are cultured in the absence of methotrexate, a *DHFR* inhibitor [84]. Remarkably, this process can be augmented using specific drugs such as low-dose hydroxyurea or dimethyl sulfoxide. When low doses of hydroxyurea are employed, the percentage of spontaneously differentiated cells is increased and the encapsulation of *MYC* amplifications within micronuclei is concurrently reduced [85]. MicroDNAs, found to be significantly increased in cancer cells, are small circular DNA molecules capable of being transcribed into functional microRNA and novel short hairpin or small interfering RNA, even without a canonical promoter [86]. These findings not only underscore the potential therapeutic avenues for targeting eccDNA-mediated genomic instability but also emphasize the translational relevance of such strategies in mitigating the aggressive characteristics of cancerous lesions.

## 7. Noninvasive Diagnostic Potential of eccDNA

EccDNA exhibits unique properties which enable its passage through the eukaryotic cell membrane [87]. This characteristic renders eccDNA highly promising in liquid biopsies. However, the exact mechanism underlying the release of cfDNA remains unclear, with postulations suggesting that it could be a consequence of genomic instability [88]. Recently, the detection of human eccDNA in the bloodstream has opened new possibilities for its application in liquid biopsies [89]. Xu et al.’s research further underscores the potential of eccDNA as a novel serum biomarker for the early diagnosis and prognosis assessment of lung adenocarcinoma [90].

The quantity of eccDNA in plasma is indeed influenced by the initial DNA input, and the detection of eccDNA is also related to the enrichment efficiency of circular DNA [32]. This suggests that the levels of eccDNA observed in plasma samples may be the result of multiple factors, including the process of cell-released DNA and subsequent experimental processing steps. During the progression of normal hematopoiesis and acute myeloid leukemia (AML), there is typically a rise in the abundance of eccDNA, suggesting a potential association between eccDNA and the pathophysiological processes of AML [91]. Lo’s discovery of circulating fetal DNA in maternal plasma holds implications for non-invasive prenatal diagnosis [92], as further evidenced by Sin et al.’s observation of eccDNA in maternal plasma [93]. They found that eccDNA molecules in plasma are typically longer than their linear counterparts. In addition to cancer and pregnancy-related diseases, eccDNA is also present in the serum of diabetic patients [94].

Similar to linear cfDNA, eccDNA may be released through various mechanisms such as cell secretion, apoptosis, necrosis, cell lysis, and the rupture of nuclear budding and micronuclei, occurring in both normal and diseased tissues, as well as the direct lysis of circulating cells [95,96]. Compared to linear DNA, eccDNA demonstrates a remarkable level of structural stability, rendering it an ideal source for potential biomarkers [93]. Numerous studies have indicated that extrachromosomal DNA elements present in maternal plasma hold promise as candidate biomarkers for diagnosing and monitoring various diseases [97]. The findings of Hansen et al. set a benchmark for eccDNA purification methods and have prompted inquiries into the optimal requirements for achieving the fast and sensitive detection of SNP mutations on eccDNA, surpassing the sensitivity of primer-based qPCR detection [98]. The elucidation of mechanisms governing the release of eccDNA and its potential as a diagnostic tool underscores its significance in clinical research and its potential to revolutionize disease management and treatment.

EccDNA presents distinct advantages over linear circulating cfDNA as a biomarker [99]. Its closed circular structure not only enhances resistance to nuclease digestion but also provides a higher level of stability, thereby increasing the possibility of accurate detection in complex biological environments [32]. Additionally, certain eccDNA identified in the circulation, such as microDNA, possess lengths far exceeding those of linear DNA, which not only facilitates detection but also enables dynamic monitoring, offering richer information on disease progression [89]. It is noteworthy that eccDNA demonstrates lineage and cell type specificity across different cells, allowing it to reflect the specific status of cells or tissues more accurately, providing precise guidance for disease diagnosis and treatment [100].

However, despite the enormous potential of eccDNA as a cancer biomarker, its practical application still faces challenges. These include enhancing the sensitivity and specificity of detection methods, improving data analysis techniques, and devising effective elimination strategies. For instance, the abundance of eccDNA varies significantly across different diseases, potentially resulting in undetectable quantities in certain disease states [101]. Currently, there is a lack of reliable and direct methods to quantify the abundance of circular DNA elements [48], and there is no universally accepted gold standard for analyzing eccDNA [102].

**Figure 4:**
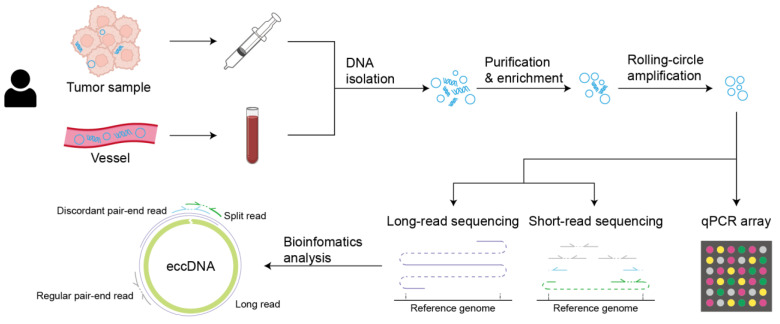
An illustration of technical pipeline in clinical oncology.

The proposed standardized technical pipeline for clinical oncology entails the initial step of obtaining tumor samples through either a tissue biopsy or blood extraction from the vasculature. Subsequently, DNA isolation, purification, and enrichment procedures are conducted to isolate eccDNA. Following this, the eccDNA undergoes rolling-circle amplification, followed by long-read or short-read sequencing to elucidate its structural characteristics. Additionally, certain oncogenes harbored within the eccDNA can be detected using qPCR arrays. Figure 4 provides a succinct illustration of this workflow. This comprehensive approach is envisaged to streamline the detection and characterization of eccDNA in clinical oncology, facilitating enhanced diagnostic and prognostic capabilities in cancer management.

## 8. Conclusions

This comprehensive review provides an in-depth overview of the functions of eccDNA as a cancer biomarker, delving into its mechanistic roles in tumor progression and diagnostic potential. EccDNA has been unequivocally established to play a pivotal role in the onset and progression of diseases, breaking through the traditional boundaries and isolation methods of genetics, thereby offering a fresh perspective for understanding the essence of diseases. Looking ahead, we firmly believe that with research advances, the mysterious veil surrounding eccDNA will be further drawn back. This will not only deepen our comprehension of the role of eccDNA in complex diseases like cancer but also offer valuable new insights and methodologies for clinical diagnosis and treatment.

The extent to which systemic and chronic co-morbidities, as well as factors such as sex, age, and ethnicity, influence the profile of eccDNA remains largely unexplored. Further investigation into these variables is needed to elucidate their impact on the dynamics and characteristics of eccDNA in various biological contexts. It remains a critical question whether eccDNA isolated from liquid biopsies encapsulates the entirety of the genetic and prognostic data obtained from corresponding tissue biopsies. Further investigation is necessary to compare our existing knowledge concerning quantities, size profiles, and other genomic attributes between tissue and liquid biopsy samples. Moreover, additional insights could be gained by stratifying analyses based on different body fluids, including blood, cerebrospinal fluid, urine, and others, such as ascites, within the category of cf-DNA. This comprehensive approach will enhance our understanding of the diagnostic and prognostic utility of eccDNA across diverse biological matrices.

To achieve this goal, collaboration between academia and industry is imperative to drive continuous advancements in eccDNA-related research. Through ongoing innovation in research methods and technologies, we aspire to detect and analyze eccDNA more accurately, thereby developing more effective strategies for cancer diagnosis and treatment. Moreover, with the integration of technologies such as big data and artificial intelligence, the study of eccDNA will enter a new phase of development, paving the way for more precise and personalized diagnostic and therapeutic approaches for cancer patients. In conclusion, as the role of eccDNA as a disease biomarker is becoming increasingly evident, further research and collaboration among interdisciplinary teams are essential to overcome the technical obstacles and realize the full potential of eccDNA as a valuable biomarker in cancer diagnosis, prognosis, and treatment monitoring.
